# Chinese insurance agents in “bad barrels”: a multilevel analysis of the relationship between ethical leadership, ethical climate and business ethical sensitivity

**DOI:** 10.1186/s40064-016-3764-2

**Published:** 2016-12-05

**Authors:** Na Zhang, Jian Zhang

**Affiliations:** 1 School of Economics and Management, Beihang University, No. 37 Xueyuan Road, Haidian District, Beijing, 100191 China; 2 Donlinks School of Economics and Management, University of Science and Technology Beijing, No. 30 Xueyuan Road, Haidian District, Beijing, 100083 China

**Keywords:** Business ethical sensitivity, Ethical leadership, Ethical climate

## Abstract

**Background:**

The moral hazards and poor public image of the insurance industry, arising from insurance agents’ unethical behavior, affect both the normal operation of an insurance company and decrease applicants’ confidence in the company. Contrarily, these scandals may demonstrate that the organizations were “bad barrels” in which insurance agents’ unethical decisions were supported or encouraged by the organization’s leadership or climate.

**Objective:**

The present study brings two organization-level factors (ethical leadership and ethical climate) together and explores the role of ethical climate on the relationship between the ethical leadership and business ethical sensitivity of Chinese insurance agents.

**Results:**

Through the multilevel analysis of 502 insurance agents from 56 organizations, it is found that organizational ethical leadership is positively related to the organizational ethical climate; organizational ethical climate is positively related to business ethical sensitivity, and organizational ethical climate fully mediates the relationship between organizational ethical leadership and business ethical sensitivity.

**Conclusions:**

Organizational ethical climate plays a completely mediating role in the relationship between organizational ethical leadership and business ethical sensitivity. The integrated model of ethical leadership, ethical climate and business ethical sensitivity makes several contributions to ethics theory, research and management.

## Background

The insurance industry plays an important role in China’s economy and contributes to the economy’s risk management. The insurance industry has made significant achievements since China’s reform and government de-regulation; however, the society’s image of the insurance industry in China remains negative. Unethical behavior, such as twisting meanings, misrepresenting or misleading customers, and rebating frequently occur in China’s current insurance industry. The moral hazards and poor social image arising from insurance agents’ unethical behavior impact the normal operation of the insurance company and decrease insurance applicants’ confidence (Fu and Deshpande 2014).

Many instances of corporate misconduct originate from employees’ distortion of commercial behavior for their own purposes. Rest (1986) created the phrase “ethical sensitivity”, which highlights the ethical aspects of the situation. This concept involves identifying different possible steps of action and the ways the choice of an action will affect concerned parties. Awareness of the ethical issues of various work situations is the first of four components to make an ethical decision and follow ethical behavior. Without identifying the ethical aspects of the situation, it is impossible to solve any ethical problem because if there is no initial recognition, there is no problem (Treviño and Brown 2004). The severity of the insurance industry’s current situation has made the study of insurance agents’ business ethical sensitivity an urgent matter.

What affects insurance agents’ recognition of ethical issues at work? Previous research in ethical decision-making has not solved the problem. Although studies have examined some influences of demographic factors, cognitive-personality factors, work-organization factors, and situational factors on ethical sensitivity, the relationships between these different level factors have not been previously discussed, as noted in O’Fallon and Butterfield’s review (O’Fallon and Butterfield 2005). Other studies have analyzed how person-organization fit (an individual variable) interacted with ethical culture (an organizational variable) to predict the ethical intention of employees in insurance and financial organizations (Ruiz-Palomino and Martínez-Cañas 2014). However, when there are considerably fewer articles in the literature that analyze ethical sensitivity as the dependent variable (Collins 2000). Particular importance should be attributed to the interrelationship of different level factors affecting individuals’ business ethical sensitivity, which has not been addressed in existing research.

In China’s fiercely competitive insurance market, there are faulty mechanisms in most companies’ promotion systems; anyone who has a high individual performance and recruits more new agents will be promoted. However, if an unethical insurance agent is promoted to run larger groups, his unethical behaviors will be demonstrated to his subordinates and lead to the development of an unethical culture in the company.

Yet prior empirical work on ethical leadership has primarily focused on direct relationships between leader behaviors and the responses of their immediate followers (Yammarino and Dansereau 2008). Researchers have not comprehensively tested the path through which upper-level leaders influence the cognitions of lower-level followers. According to Treviño and Youngblood’s “bad barrels” theory (1990), subordinates give into organizational influence to comply with company transgressions, and their unethical behavior is linked to an immoral organizational culture. It is unclear whether the organizations were “bad barrels,” in themselves, and whether the insurance agents’ unethical decisions were supported or even encouraged by the organization’s leadership or climate. Thus, currently little is known about how ethical leadership and contextual factors at higher organization levels influence outcomes at lower levels.

The present study brings together two organization-level factors (ethical leadership and ethical climate) and explores the relationship between these variables and the business ethical sensitivity of Chinese insurance agents. Testing this integrated model makes several contributions to ethics theory. On one side, although there is increasing interest in ethical leadership, the majority of research has focused on unethical behaviors (Elçi et al. 2013; Mayer et al. 2010), whereas we examined employees’ business ethical sensitivity as an outcome, which is the first psychological component of ethical decision making and is the basic logical starting point of all kinds of ethical behaviors. On the other side, there is little empirical support for the underlying mechanism linking upper-level organization’s ethical leadership to lower-level employee cognitions. To address this gap in the literature we provide a theoretical model for linking upper-level ethical leadership to lower-level employee business ethical sensitivity through upper-level ethical climate.

## Theoretical framework and hypotheses

### Business ethical sensitivity

Rest (1986) developed the most widely used model of ethical decision-making. He built the four-component model by working backwards from his observations and concluded that incorporating an ethical dimension in one’s decisions and behaviors was the result of four distinct sequential processes: (1) ethical sensitivity, (2) ethical judgment, (3) ethical intention, and (4) ethical action. Ethical sensitivity,^1^ which he defined as recognizing the presence of an ethical issue, is the first step in ethical decision-making because we cannot solve a moral problem unless we first know that one exists. Hébert et al. (1990, p. 141) defined ethical sensitivity as, “the ability to recognize ethical issues”. Shaub (1989, p. 61) described ethical sensitivity as “the ability to recognize that a situation has ethical content when it is encountered”. Commonalities in these definitions involve the recognition and ability to perceive an ethical issue that can have an impact on others.

Although the two terms have occasionally been used interchangeably (Jordan 2007, 2009), ethical sensitivity is distinct from “ethical awareness” (Reynolds 2006). Reynolds (2006, p. 233) described moral awareness as “the determination that a situation contains moral content” and observed that ethical sensitivity was the individual’s ability to achieve moral awareness to make a distinction from moral awareness (Reynolds 2008). As ethical sensitivity pertains to an individual’s ability to discern ethical issues from other issues in context while ethical awareness is the decision to consider an identified issue as truly moral in nature (Sparks 2015), the constructs differ sufficiently to consider them distinct from each other. In this study, we considered business ethical sensitivity to be the ability to recognize that a work situation has ethical content when it is encountered. Applied business ethical sensitivity is the act of recognizing and identifying the ethical aspects of work situations, which is the basic logical starting point of ethical behaviors.

The existing research of business ethical sensitivity has mainly focused on the marketing, auditing and accounting professions. Hunt and Vitell (1986) proposed a general theory of marketing ethics, which broadly evaluates ethical behaviors based on how cultural, organizational, and industrial factors interact with individual factors to shape perceptions, which in turn impact judgment, intentions, and behaviors. Marta et al. (2008) investigated how a marketer’s personal religiousness, relativism, and ethical values influence ethical sensitivity in hypothetical marketing scenarios, and the results showed significant differences between Mexican and U.S. marketers on these variables. In addition, Yetmar and Eastman (2000) investigated whether role ambiguity and role conflict were negatively associated with ethical sensitivity, and whether job satisfaction, ethical orientation, and professional commitment were positively associated with ethical sensitivity using tax practitioners. However, their results only supported the role conflict and job satisfaction’s influence on tax practitioners’ ethical sensitivity. While Shaub et al. (1993) studied the effects of professional commitment, organizational commitment and ethical orientation on auditors’ ethical sensitivity; they found only a negative relationship between auditors’ ethical orientation and ethical sensitivity. Uyar et al. (2015) found that seniority (experience, income level, and title) and religiosity in the accounting profession had a positive influence on ethical sensitivity; and within the theoretical approaches, deontology had a positive influence on ethical sensitivity, whereas egoism had a negative one.

Ethical sensitivity, defined as an individual’s ability to recognize the ethical content of the decision-situation, serves as a type of triggering mechanism that begins the ethical decision-making process (Sparks and Merenski 2000). As Rest (1986) argued that all four stages were conceptually different and that success in one stage did not mean success in other stages, Johari et al. (2011) identified significant positive relationships between the first three processes of ethical decision-making among auditors. Musbah et al. (2016) reported a significant relationship between ethical recognition and ethical judgment and also between ethical judgment and ethical intention, but ethical recognition did not significantly predict ethical intention, and the same results were found in Haines et al.’s (2008) research. Similarly, Jagger (2011) found that levels of ethical sensitivity had a significant impact on the development of moral judgment. On the contrary, Valentine and Fleischman (2003) reported that recognition of the ethical issue was unrelated to ethical judgment. One reason for the absence of a clear one-to-one relationship between ethical sensitivity, judgment, intention and behavior in these studies (Pedersen 2009) may be that only the main effects were addressed, with limited focus on crucial moderating and mediating influences. Thus, there is a wide variety of moderators and mediators of ethical decision-making that need to be investigated to better understand the process, and ethical sensitivity should be an initial focus of ethical decision making.

### Ethical leadership

According to Brown et al. (2005, p. 120), ethical leadership is “the demonstration of normatively appropriate conduct through personal actions and interpersonal relationships, and the promotion of such conduct to followers through two-way communication, reinforcement, and decision-making.” Ethical leaders signal to the staff that doing the right thing is encouraged and valued; in return, employees are more inclined to think that the organizational environment is ethical (Mayer et al. 2010).

A social learning perspective on ethical leadership recommends that leaders influence followers through modeling ethical behavior (Bandura 1977). The modeling contains a wide range of psychological matching processes, including observational learning, imitation, and identification. It also notes that people are likely to take notice and emulate actions from trusted and attractive role models. In addition to direct observation, employees are affected by their supervisor indirectly, as the supervisor has the power to punish or reward them for their ethical or unethical behavior.

A growing number of empirical researches have shown that ethical leadership enhances employee ethical behavior, such as voice behavior (Walumbwa and Schaubroeck 2009) and organizational citizenship behavior (Ruiz-Palomino et al. 2011). In addition, scholars find that ethical leadership is effective in reducing followers’ unethical behavior, such as misconduct (Mayer et al. 2010), deviant behavior (Avey et al. 2011), and organizational bullying in the workplace (Stouten et al. 2010).

Since people are social beings who are affected from other people, leaders are the important role models in order to foster a favorable ethical climate (Elçi et al. 2013). Essentially, ethical leaders help create an organizational climate in which doing the right thing is expected and valued through role modeling appropriate behavior. According to Stringer (2002), many studies have revealed that the single most important determinant of an organization’s climate is the daily behavior of the organization’s leaders. For example, Neubert et al. (2009) suggested that ethical leadership can virtuously influence organizational members’ perceptions of the ethical climate. Grojean et al. (2004) argued seven mechanisms by which leaders convey the importance of moral values to the staff, and establish expectations regarding ethical behavior that becomes entrenched in the organization’s climate. Moreover, they found that these mechanisms influence employees’ practices and expectations, further improving the salience of ethical values and generating shared awareness that, eventually, form an organization’s climate. In addition, Dickson et al. (2001) showed that the key factor of an organization’s ethical climate is the leader’s ethical behavior. According to Schein (1985), Sims and his colleagues (2000; Sims and Brinkman 2002) discussed how leaders shape and strengthen the organization’s ethical climate, and Logsdon and her colleagues had a similar view (Logsdon and Yuthas 1997; Logsdon and Corzine 1999). Consequently, we expect ethical leadership to have a positive impact on both the organization’s ethical climate and the business ethical sensitivity of Chinese insurance agents. Specifically, we predict that:

#### **H1**

Organization-level ethical leadership positively relates to insurance agents’ business ethical sensitivity.

#### **H2**

Organization-level ethical leadership positively relates to an organization’s ethical climate.

### Ethical climate

Taking the lead from Schneider’s definition of the work climate (1983) and employing a broad based definition of ethics, Victor and Cullen (1988, p. 51) developed the concept of an organizational ethical climate, which they defined as “the shared perceptions of what ethically correct behavior is and how ethical issues should be handled”. The ethical climate not only affects which issues organizational members consider to be ethics-related, but it also determines the ethical standards members use to understand, weigh, and resolve such issues (Martin and Cullen 2006). Cullen et al. developed 36 ethical climate descriptions based on type of ethical values (egoism, benevolence, or principle) and locus of analysis (individual, local, or cosmopolitan; Cullen et al. 1993). Five climate types emerged from their factor analysis: caring, rules, instrumental, independence, and law and code (Victor and Cullen 1988), and formed the most well-known classification of ethical climate (Shin 2012).

As perceptions of organizational climate may vary within a firm and different subunits or work groups may possess different climates (Victor and Cullen 1987, 1988), in the current study, ethical climate was conceptualized as an organizational-level construct that represents insurance agents’ shared perceptions of the ethical climate of the sales departments.

The present study focuses only on law and code and rules dimensions of ethical climate, because the law and code and rules dimensions best reflect the essence of ethical climate compared with other three dimensions. Researchers who considered ethical climate as a uni-dimensional concept defined ethical climate as employees’ perceptions of the presence of a code of ethics, corporate policies on ethics, and top management actions with regard to ethics, such as in Jamarillo et al. (2006) and Schwepker Jr. (2001). This conceptualization of ethical climate was captured in law and code and rules dimensions. In addition, Leung (2008) categorized rules and law and code dimensions as higher levels of ethical climate than the other dimensions. Consequently, the operational definition of ethical climate based on Victor and Cullen’s (1988) law and code and rules dimensions was insurance agents’ shared perceptions of ethical policies, practices, and procedures within the organization.

Many researchers have discussed the relationship between ethical climate and ethical sensitivity. For example, Victor and Cullen (1988) noted that the predominant type of the organization’s ethical climate may affect the types of ethical conflicts considered, a short leap to “recognition”. Wyld and Jones (1997) show that the ethical climate does not influence just the final behavior, but the decision-making process, from issue recognition (or non-recognition) to decision. The results of the research by VanSandt et al. (2006) demonstrated that ethical work climate is a major factor of a person’s ethical awareness. In addition, Cohen (1995) suggested that the organization’s ethical climate is used to address issues with an ethical component, and these issues include recognizing ethical issues.

Consequently, many researchers (Alteer et al. 2013; Lützén et al. 2010; Schluter et al. 2008) have admitted the possible relationship between the organization’s ethical climate and the individual’s ethical sensitivity; based on that, we will examine the literature related to the business ethical sensitivity of Chinese insurance agents and propose that:

#### **H3**

An organization’s ethical climate positively relates to insurance agents’ business ethical sensitivity.

In previous studies, results have shown that leaders have substantial power to build and maintain ethical norms and processes and to create a particular type of ethical climate. As noted above, Wimbush and Shepard (1994) found that supervisors “influence employees’ perceptions of the policies and practices, i.e., ethical climate” (p. 645). Schminke et al. (2005) suggested that organizational leaders play an important role in the organization’s ethical climate. Further, Carpenter and Reimers (2005) found that managers’ attitudes, shaped by the tone set by top executives, significantly influence managers’ decisions to behave unethically. Many researchers investigated the effects of both ethical leadership and ethical climate on employees’ antisocial behaviors (Elçi et al. 2013), misconduct (Mayer et al. 2010), attitudes toward corporate social responsibility (Choi et al. 2015), and organizational commitment (Constandt et al. 2016). Ethical sensitivity, which is the basic logical starting point of all types of ethical or unethical behaviors, will also be affected by the interrelationship of ethical leadership and ethical climate. Overall, ethical leadership relates positively to individual’s ethical sensitivity through ethical climate. In other words, ethical climate is the mechanism by which ethical leadership relates positively to ethical sensitivity. Accordingly, it is hypothesized that:

#### **H4**

An organization’s ethical climate is a mediator between organization-level ethical leadership and insurance agents’ business ethical sensitivity.

**Fig. 1:**
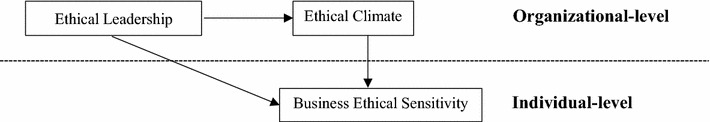
Overall conceptual framework

Figure 1 provides an overview of the conceptual framework for this study. A review of prior literature indicated that business ethical sensitivity can be affected by the interrelationship of ethical leadership and ethical climate. The present study brings the two organization-level factors together and explores the mediation effects of ethical climate between the ethical leadership and business ethical sensitivity of Chinese insurance agents.

## Methods

### Sample

The respondents consisted of 600 insurance agents from 65 organizations from the PICC Life Insurance Company Ltd. in the Shijiazhuang, Baoding and Handan regions. In PICC Life Insurance Company Ltd., anyone who has a high individual performance and recruits more than three new agents will be promoted as agency supervisor, and they will form a sales department. In this study, the 65 organizations were all sales departments. 81 surveys with missing values were dropped at first, and then to alleviate aggregation biases, questionnaires from organizations with fewer than 3 respondents were excluded from the sample. Thus, other 17 agents in 9 organizations from the sample were dropped again. The final sample was including of 502 insurance agents from 56 organizations. The 56 organizations varied in size and region. The average size of the organizations in PICC Life Insurance Company Ltd. was 15 insurance agents. On average, 9 insurance agents per organizations participated in the study. 19 organizations were from Shijiazhuang regions, 21 organizations were from Baoding regions and 16 organizations were from Handan regions. Of the 502 participants, 35.1% were men and 64.9% were women. Age was measured in 4 bands to preserve anonymity of the participants. The sample in Band 2 (31–40 years) and in Band 3 (41–50 years) was nearly equal. In the sample, 63.7% had a high school education or lower. Similar to age, experience in the insurance industry was measured in bands, 5 in this instance, and 32.1% of the sample was in Band 2 (3–5 years).

The consent forms, which indicated that participation was voluntary and ensured data anonymity, were distributed to the insurance agents. After they read, signed and returned it, the survey instrument was distributed to them. The questionnaires took approximately 20 min to complete and included the psychological measures and demographic information of each participant. They completed the survey during staff meeting times.

### Measures

The comprehensive survey covered a variety of items, including questions about business ethical sensitivity, ethical leadership, ethical climate, and demographic variables. All the measures were prepared in Chinese. However, the ethical leadership and ethical climate scale were cited in English articles and subsequently translated into Chinese by the second author. To avoid distortion in translation, the scales were translated back into English by two professionals independently and compared with the original English version of the paper.

### Business ethical sensitivity

Chinese insurance agents’ business ethical sensitivity (CIABES) was measured using a custom-made questionnaire (Zhang and Zhang 2016). Although scales of ethical sensitivity have been developed in many domains, such as teaching (Gholami et al. 2015), marketing (Sparks and Hunt 1998), and nursing (Kim et al. 2002), the CIABES scale is the only instrument for measuring ethical sensitivity in the insurance industry. As Sparks (2015) noted that ethical sensitivity was context specific, many ethical issues were relevant only to certain situations or settings, and much of the empirical literature on ethical sensitivity focused on professional settings. Especially, in the scale of general employees’ ethical sensitivity used in the previous studies (Hadjicharalambous and Walsh 2012; Stevens et al. 1993), the dimensions of personal use and the padding of expenses were not suitable for the insurance agents. The CIABES scale we used consisted of four dimensions: Cheating the company, Misleading customers, Providing false information, and Launching personal attacks, which were all context specific to the insurance profession. As presented in the “Appendix”, the survey included 15 items using a five-point Likert scale with possible scores of 1 (strongly agree), 2 (agree), 3 (indifferent), 4 (disagree) and 5 (strongly disagree). The descriptions used in the scale differed from those used in instruments in the past, which ranged from “very ethical” to “extremely unethical.” The much more concealed responses of “strongly agree” to “strongly disagree” were adopted to control for social desirability bias. High scores implied great business ethical sensitivity, and low scores indicated poor business ethical sensitivity. The overall reliability of the questionnaire was sufficiently high (α = 0.787).

The absolute validity of the four-factor model of CIABES was tested. All fit indices satisfied the requirements: χ^2^/*df* was 4.705, RMSEA was 0.079, GFI was 0.964, and CFI was 0.942. Based on the above indicators, the structure of the CIABES was acceptable, and its construct validity was good.

### Organizational ethical leadership

Brown et al. (2005) proposed social learning theory as a theoretical basis for understanding ethical leadership and developed a 10-item instrument to measure the concept. The 10 items were rated on a five-point scale (5 = strongly agree; 1 = strongly disagree). For example, “My superior makes fair and balanced decisions”. High scores implied higher ethical leadership. Cronbach’s α for ethical leadership was 0.932.

To measure the organization’s ethical leadership, we aggregated the ethical leadership measures of individual insurance agents from each organization using the direct consensus composition approach (Chan 1998). As all the samples were insurance agents and the agency supervisors were not included, the agents in one organization have the only one and the same superior. Then the average ethical leadership measures of all the respondents in an organization represented the ethical leadership of the organization. The within-group agreement (r_wg_) for ethical leadership for each organization was calculated to justify the aggregation (James et al. 1993). For the 56 organizations, the mean of their r_wg_s was 0.978 for ethical leadership, which was higher than the generally acceptable value of 0.70 (James et al. 1993), indicating a reasonable level of agreement. Additionally, ICCs were calculated. The ICC(1) coefficient represents the proportion of variance in ratings at the individual level that is attributed to group membership, whereas the ICC(2) coefficient represents the reliability of the group-level means (Chen et al. 2012). The ICC(1) coefficient was 0.199, and the ICC(2) coefficient was 0.938 for ethical leadership. The aggregation of individual-level measures of ethical leadership into the organization’s ethical leadership measure was acceptable.

### Organizational ethical climate

Followed Shin’s (2012) study, the ethical climate scale consisted of four items from Victor and Cullen’s (1988) Law and Code Climate Scale and four items from their Rules Climate Scale. The insurance agents were asked to rate the ethical climate they perceived in the workplace on a five-point Likert scale, with 1 representing “completely false” and 5 representing “completely true.” For example, “It is important to follow rules and procedures in this organization”. In the current study, the scale’s α reliability was 0.824.

To measure the organization’s ethical climate, we also aggregated the ethical climate measures of individual insurance agents from each organization using the direct consensus composition approach (Chan 1998).The average ethical climate measures of all the participants in an organization represented the organization’s ethical climate. The mean of 56 organizational r_wg_s was 0.968, the ICC(1) coefficient was 0.083 and the ICC(2) coefficient was 0.835 for ethical climate, which proves that aggregating the individual-level measures of ethical climate into the organization’s ethical climate measure was explainable.

## Results

### Descriptive statistics

Descriptive statistics are given in Table 1. The means, standard deviations, and coefficient alphas of demographic variables, ethical leadership, ethical climate and business ethical sensitivity are listed at both the individual and the organizational levels.

**Table 1:** Descriptive statistics

Variables	M	SD	1	2	3	4	5	6
Individual-level (n = 502)								
1. Gender	0.32	0.20						
2. Experience in insurance industry	1.77	1.09	−.078					
3. Age group	2.30	0.61	−.084	0.838***				
4. Education level	2.38	0.54	−.070	0.271***	0.321***			
5. Business ethical sensitivity	3.96	0.42	0.149**	0.065	−.033	−.019		
Organizational-level (n = 56)								
6. Ethical leadership	4.46	0.28						
7. Ethical climate	4.31	0.22						0.642***

* p < 0.05; ** p < 0.01; *** p < 0.001 (2-tailed)

### Hypothesis tests

As the data in the present research were multilevel in nature, with organization’s ethical leadership and ethical climate measured at the organizational level (level 2), and individual business ethical sensitivity measured at the individual level (level 1), a suitable analysis method was needed to consider the hierarchical structure of the data. As a result, hierarchical linear modeling (HLM; Raudenbush and Bryk 2002) was conducted, which followed the logic of traditional mediation analyses (Baron and Kenny 1986), and yet extended the chain of relationships to potentially include cross-level effects (Krull and MacKinnon 1999, 2001).The computer program HLM 6.08 was used, with the restricted maximum likelihood (RML) estimation method testing the hypotheses.

In a multilevel model, centering can help with interpreting contextual effects and cross-level interactions and in examining random regression slopes (Wu and Wooldridge 2005). Grand mean centering is normally used unless there is a clear theory (or empirical rationale) supporting the priority of individuals’ relative group standings in relation to the dependent variable, in which case group mean centering is preferred (Chu et al. 2014). According to Hofmann and Gavin (1998), grand mean centering techniques have been recommended for testing both direct and cross-level meditational effects. In our research, the antecedent and mediator are both measured at level 2 and the dependent variable at level 1, which is labeled as 2-2-1 model, and there are no level 1 relationships that could interfere with the estimation of level 2 mediation effects. Thus, variables were entered into the models using grand mean centering.

To demonstrate the between-organization hypotheses, there must be a significant between-group variance in insurance agents’ business ethical sensitivity. Consequently, a null model was first run to examine whether there was systematic between-group variance in insurance agents’ business ethical sensitivity. The significant Chi squares for business ethical sensitivity were χ^2^(55) = 158.867, *p* < 0.001, τ00 = 0.033, σ^2^ = 0.149, ICC(1) = 0.181. ICC(1) value was greater than 0.06, indicating that appropriate variance in individual-level variables existed between-group, and justified further the HLM analysis (Bryk and Raudenbush 1992).

For the mediation tests, we used Mathieu and Taylor’s (2007) approach, which applies the mediation rules of evidence outlined by Baron and Kenny (1986), and estimated the following steps: first, evaluate the significance of independent and dependent variable relations. Second, test the influence of the independent variable on the mediator. Third, test the relationship between the mediator and the dependent variable. Fourth, any significant relationship between the independent and dependent variables becomes non-significant or weaker when the mediator is added.

Our results were organized in terms of the steps listed above. Hypothesis 1 predicted that, within an organization, ethical leadership would positively relate to insurance agents’ business ethical sensitivity. As shown in Model 2 of Table 2, the findings indicated a significant positive relationship between organizational ethical leadership and business ethical sensitivity (γ = 0.215, *p* < 0.05), supporting hypothesis 1.

**Table 2:** HLM analyses

Variables	Ethical climate	Business ethical sensitivity		
	Model 1	Model 2	Model 3	Model 4
Intercept Level 1: Individual-level (n = 502)	2.067***	3.972***	3.973***	3.972***
Gender				
Experience in insurance industry				
Age group				
Education level				
Level 2: Organization-level (n = 56)				
Ethical leadership	0.502***	0.215*		−.048
Ethical climate			0.493***	0.533**
R^2^	0.412	0.060	0.275	0.253

* p < 0.05; ** p < 0.01; *** p < 0.001 (2-tailed)

Hypothesis 2 suggested that organization-level ethical leadership positively relates to organizational ethical climate. As shown in Model 1 of Table 2, strong support was shown for hypothesis 2, with the organizational ethical leadership significantly positively related to ethical climate (γ = 0.502, *p* < 0.001).

Hypothesis 3 predicted that organizational ethical climate would positively relate to insurance agents’ business ethical sensitivity. As shown in Model 3 of Table 2, the study findings indicated a significant positive relationship between organizational ethical climate and business ethical sensitivity (γ = 0.493, *p* < 0.001). Therefore, hypothesis 3 was supported.

Hypothesis 4 suggested that organizational ethical climate was a mediator between organization-level ethical leadership and insurance agents’ business ethical sensitivity. As is shown in Model 4 of Table 2, organizational ethical climate was significantly positively related to business ethical sensitivity (γ = 0.533, *p* < 0.001), but ethical leadership was not related to business ethical sensitivity (γ = −0.048, *p* > 0.05). As the independent variable had no effect when the mediation was controlled, the full mediation holds (Baron and Kenny 1986). Indeed, a Sobel test of the cross-level mediated effect was significant (Sobel = 3.659, *p* < 0.001).Thus, support was given for hypothesis 4, and the fully mediating effect of organizational ethical climate on the relationship between organizational ethical leadership and business ethical sensitivity has been validated.

## Discussion

### Findings

To combine research in psychology and management and to expand the research field of insurance agents, the aim of this study was to investigate the relationship among organization’s ethical leadership, ethical climate and the business ethical sensitivity of Chinese insurance agents.

We found evidence of a direct link between organizational ethical leadership and business ethical sensitivity; the presence of ethical leaders in the organizations positively related to insurance agents’ business ethical sensitivity. In line with the study’s hypotheses, findings showed that organizational ethical leadership has effects on the ethical climate, which means that leaders with their own principles and behaviors shape the ethical atmosphere in their organizations. This finding is consistent with Dickson et al. (2001) and Neubert et al. (2009). The findings of our study also suggested that by creating an ethical environment within the organization, it is possible to help employees enhance their recognition of ethical issues. This finding is consistent with Key (2002), VanSandt et al. (2006) and Schminke et al. (2005), who view that an ethical work climate is a primary predictor of individual moral awareness.

What is important is that we verified a mediator of organizational ethical leadership’s influence on employees’ ethical sensitivity. Although some empirical studies have tested the relationship between ethical leadership and employees’ behavior, we currently do not have a solid understanding of the process underlying the relationship between ethical leadership and employees’ ethical sensitivity. In this research, we find that organizational ethical climate mediates the relationship between the organization’s ethical leadership and the insurance agents’ business ethical sensitivity. Therefore, we proved that when the leaders are ethical, they are more likely to create a circumstance in which doing the right thing is expected and valued (Brown et al. 2005). Ethical circumstances include the staff’s consciousness of ethical problems and desires to maintain high moral standards. When employees work in an ethical climate, they are more inclined to be sensitive to ethical issues in various situations. These results are consistent with the previous research on this topic. Previous research findings indicate a positive relationship among ethical leadership and organization members’ perceptions of their organization’s ethical climate.

Additionally, this study contributes to the issue-contingent model literature. Jones (1991) suggests that organizational factors are likely to play a role in ethical decision making at the last two steps: establishing moral intent and engaging in moral behavior. We expanded his research scope and confirmed that organizational ethical leadership and climate affect ethical sensitivity, which is the first step in ethical decision-making.

### Implications for management

The findings of this research have a number of important implications for managers in a variety of organizations, especially for managers in insurance companies.

First, given the benefits of ethical leadership in developing an ethical climate, insurance companies should pursue choosing and/or training ethical leaders to increase insurance agents’ business ethical sensitivity. Especially in the promotion process, they should use selection tools that test integrity, ethical standards, and concern for the others. Organizations should also invest in ethics training for leaders. Accordingly, through focusing on selection and training, the organization can help to improve ethical leadership. Then, the manager should become a role model for ethical behavior, communicating regularly about ethics and values and using the punishment/reward system to hold everyone in compliance with the company’s values and standards.

Second, the study suggests that ethical climate is an important antecedent to insurance agents’ business ethical sensitivity. An ethical climate serves an important role in helping staff members know how to address ethical issues. Emphasizing the value of being an ethical employee is critical for practices, policies, and procedures. Human resource management practices should be highly visible so that insurance agents learn not only from their own experiences but also vicariously through others’ rewards or punishments. These strategies are useful for improving insurance agents’ awareness of ethical issues.

In addition, business ethical sensitivity should be an initial focus of ethics education in insurance companies’ training processes; the CIABES scale should be applied to test the effectiveness of education and training on insurance agents’ business ethical sensitivity. The addition of the CIABES to the performance assessment process and rewarding advanced agents will, through assessment and social pressure, enhance insurance agents’ consciousness of ethical actions and thus regulate the unethical practices among insurance agents.

### Limitations and suggestions

Although this study contributes to the understanding of ethical leadership and ethical climate relation with the business ethical sensitivity of Chinese insurance agents, it also has some limitations.

All of the participants in the study were from three regions in Hebei province, and they were all from one domestic insurance company, PICC Life Insurance Company Ltd.; the insurance agents in other provinces or in other companies, especially in foreign insurance companies, were not investigated. The sample data used may not be sufficiently comprehensive to apply our findings to insurance companies worldwide. Future research should select more types of insurance companies by expanding the geographical scope and paying more attention to the insurance agents in first-tier cities to compare the different levels of economic development on insurance agents’ business ethical sensitivity and ethical behaviors.

In addition, all variables were measured via self-report with survey methodology, leaving open the chance for common method variance to influence the results. We acknowledge that the use of self-report measures is a potential limitation of the study. Nevertheless, we note that the most valid source for information about an individual’s ethical sensitivity is the individual himself or herself. The usage of self-report measurement tools always incorporates the problem of social desirability bias. To overcome this weakness, in the future, mutual evaluation among leaders and employees should be used to indicate more reliable information about the ethical levels of participants.

Continuing prior studies’ focus on the relationship between ethical leadership and beneficial outcomes for organization and employees (Ruiz-Palomino et al. 2013), the present research focuses centrally on the mediation effect of organizational ethical climate in the relationship between organizational ethical leadership and business ethical sensitivity. Future research could examine a wider range of the interrelationship of organizational and individual factors in relation to business ethical sensitivity. For example, researchers found that person-organization fit positively moderated the association between organizational ethical culture and employees’ ethical intent (Ruiz-Palomino and Martínez-Cañas 2014). Thus, further research should continue to test how person-organization fit could interact with organizational ethical climate and ethical leadership to explain business ethical sensitivity. Furthermore, Ruiz et al. (2015) reported that coherence among all organizational instruments (code of ethics, performance assessment system, training) most influenced the ethical intention of employees from the banking and insurance sectors. More research is needed to examine the importance of coherence in the ethics message provided within the organization for business ethical sensitivity. Finally, because of their strong ability to determine people’s ethical standards and inform their perceptions of ethical issues, cognitive-personality factors are discussed mostly in research on ethical decision-making (Craft 2013). We also recommend that researchers explore the interrelationships of work-organization factors and individual variables such as relativistic beliefs or external locus of control (Ruiz-Palomino and Bañón-Gomis 2016) to predict business ethical sensitivity.

## Conclusions

Organization’s ethical leadership was positively related to the organization’s ethical climate; organizational ethical climate was positively related to business ethical sensitivity, and organizational ethical climate mediated the relationship between organizational ethical leadership and business ethical sensitivity.

## Appendix: Chinese insurance agents’ business ethical sensitivity measure

Cheating the company:

1. Although an insurance agent knows that his client was seriously ill, he still hides the client’s information to the company and helps the client to buy the insurance product.
2. An insurance agent commits additional benefits beyond the insurance product clauses to the customer.
3. An insurance agent made a counterfeit signature for his/her client.
4. For his own benefit, an insurance agent persuades his clients to surrender and buy new products.

Misleading customers:

5. An insurance agent withholds clients’ premium or compensation.
6. An insurance agent promotes products with high commission but which do not meet the customers’ needs.
7. An insurance agent promotes products to potential customers regardless of settings and time, which interferes with their normal life.
8. An insurance agent tells his customers that their funds are unsafe in the bank, and persuades them to buy the investment managing finances life insurance products.

Providing false information:

9. An insurance agent assists his clients to provide false information to achieve the purpose of claims.
10. Although the client’s data was incomplete, the insurance agent directly made up the client’s information because he is eager to complete the order, and did not inform customer in a timely manner to modify after that.
11. An insurance agent sells products from a few different companies at the same time.
12. When an insurance agent recommends products to older clients, he does not inform the period of insurance.
13. An insurance agent opens and keeps bank book or card for collecting the premiums instead of his clients.

Launching personal attacks:

14. In order to obtain a high-quality client, an insurance agent intimidates other insurance company’s agents.
15. An insurance agent uses uncivilized words to insult a potential customer after being rejected.

## Footnotes

1 The terms moral sensitivity and ethical sensitivity are considered to be equivalent in this study, and the term ‘ethical’ is used throughout for consistency.
